# API5 confers cancer stem cell-like properties through the FGF2-NANOG axis

**DOI:** 10.1038/oncsis.2016.87

**Published:** 2017-01-16

**Authors:** K-H Song, H Cho, S Kim, H-J Lee, S J Oh, S R Woo, S-O Hong, H S Jang, K H Noh, C H Choi, J-Y Chung, S M Hewitt, J-H Kim, M Son, S-H Kim, B I Lee, H-C Park, Y-K Bae, T W Kim

**Affiliations:** 1 Laboratory of Tumor Immunology, Department of Biomedical Sciences, Graduate School of Medicine, Korea University, Seoul, Republic of Korea; 2 Department of Biochemistry, Korea University College of Medicine, Seoul, Republic of Korea; 3 Department of Biomedical Sciences, College of Medicine, Korea University, Seoul, Republic of Korea; 4 Experimental Pathology Laboratory, Laboratory of Pathology, Center for Cancer Research, National Cancer Institute, National Institutes of Health, Bethesda, MD, USA; 5 Department of Obstetrics and Gynecology, Gangnam Severance Hospital, Yonsei University College of Medicine, Seoul, Republic of Korea; 6 Institute of Women's Life Medical Science, Yonsei University College of Medicine, Seoul, Republic of Korea; 7 Graduate School of Medicine, Korea University, Ansan, Republic of Korea; 8 Department of Obstetrics and Gynecology, Samsung Medical Center, Sungkyunkwan University School of Medicine, Seoul, Republic of Korea; 9 Feinberg School of Medicine, Northwestern University, Chicago, IL, USA; 10 Immunotherapy Research Center, Korea Research Institute of Bioscience and Biothechnology (KRIBB), Daejeon, Republic of Korea; 11 Biomolecular Function Research Branch, Division of Convergence Technology, Research Institute, National Cancer Center, Goyang, Republic of Korea; 12 Comparative Biomedical Research Branch, Division of Cancer Biology, Research Institute, National Cancer Center, Goyang, Republic of Korea

## Abstract

Immune selection drives the evolution of tumor cells toward an immune-resistant and cancer stem cell (CSC)-like phenotype. We reported that apoptosis inhibitor-5 (API5) acts as an immune escape factor, which has a significant role in controlling immune resistance to antigen-specific T cells, but its functional association with CSC-like properties remains largely unknown. In this study, we demonstrated for the first time that API5 confers CSC-like properties, including NANOG expression, the frequency of CD44-positive cells and sphere-forming capacity. Critically, these CSC-like properties mediated by API5 are dependent on FGFR1 signaling, which is triggered by E2F1-dependent FGF2 expression. Furthermore, we uncovered the FGF2-NANOG molecular axis as a downstream component of API5 signaling that is conserved in cervical cancer patients. Finally, we found that the blockade of FGFR signaling is an effective strategy to control API5^high^ human cancer. Thus, our findings reveal a crucial role of API5 in linking immune resistance and CSC-like properties, and provide the rationale for its therapeutic application for the treatment of API5^+^ refractory tumors.

## Introduction

Increasing evidence suggests that a sub-population of cancer cells with stem-like properties has a prominent role in the maintenance and progression of certain cancers.^1, 2^ These rare cancer cells have been termed cancer stem cells (CSCs) and they are characterized by expression of specific cell surface markers (for example, CD44, CD133 and EpCAM),^3, 4, 5^ expression of stemness factors (for example, NANOG, OCT4 and SOX2)^6, 7^ and mammo-sphere formation in suspension culture.^8, 9^ These cells are reported to have inherently greater tumor-initiating potential, which is implicated in tumor relapse, driving primary tumor growth, as well as the seeding and establishment of metastases.^1, 2^ Therefore, targeting the CSC population may be an effective therapeutic strategy to substantially improve cancer patient survival while reducing the risk of relapse.

Previously, we developed a highly immune-resistant murine tumor cell subline, TC-1 P3, generated by serial *in vivo* selection of its immune-susceptible parental cell line TC-1 P0, which expresses the CTL target antigen, E7 of human papilloma virus 16 (HPV16).^10^ In addition to the mouse model, we also established a highly immune-resistant human tumor cell line, CaSki/D^b^ P3, generated from its immune-susceptible parental cell line CaSki/D^b^ P0 through serial selection by co-incubation of CaSki/D^b^ P0 cells, pulsed with an E7 epitope and mouse E7-specific CTLs.^11^ Interestingly, we recently found that immune selection drives the evolution of tumor cells toward a CSC-like phenotype, as well as immune resistance in both mouse and human models.^11, 12^ In the process, the transcription factor NANOG links the emergence of a stem-like state with immune escape phenotypes.^11, 12, 13^ However, it remains largely unknown what factors potentiate NANOG expression in immune-resistant cancer cells.

Apoptosis inhibitor-5 (API5), also called anti-apoptosis clone-11 (AAC-11) or fibroblast growth factor-2-interacting factor, was initially identified as an apoptosis inhibitory protein whose expression prevents apoptosis after growth factor deprivation.^14, 15^ It was suggested that API5 causes suppression of apoptosis by inhibiting caspase-3-mediated DNA fragmentation through interaction with Acinus or by negative regulation of transcription factor E2F1-induced apoptosis.^16, 17^ In addition, we demonstrated a new pathway involved in API5-mediated anti-apoptotic property that is dependent on the secretion of FGF2 and downstream FGFR1 signaling, which triggers specific degradation of the pro-apoptotic molecule, BIM, by PKCδ-dependent ERK activation.^18^ Moreover, API5 had been reported to be upregulated in multiple cancer cell lines^13^ and cancer patients,^19, 20, 21^ and to be involved in invasive potential of cancer cells.^22, 23^ Correspondingly, we had found that API5 expression was associated with pERK1/2 in a subset of cervical cancer patients and its expression predicted poor overall survival, and ectopic expression of API5 increased cell proliferation and colony formation.^19^ These observations suggest that API5 is pivotal for the development and progression of cancer, in addition to its anti-apoptotic property.

Recently, we reported that API5 acts as an immune escape factor, which has a significant role in controlling immune resistance to antigen-specific T cells both in the mouse immune-resistant model and human cancer cells,^18^ but its functional association with CSC-like properties remains largely unknown. Interestingly, API5 expression was high in CSC-enriched populations, such as immune selection-derived cells, CD44^high^ cells and sphere-forming cells. In this study, we demonstrated, for the first time to our knowledge, that API5 confers CSC-like properties, including NANOG expression, the frequency of CD44-positive cells and sphere-forming capacity. Critically, these CSC-like properties mediated by API5 are dependent on FGFR1 signaling, which is triggered by E2F1-dependent FGF2 expression. Furthermore, we uncovered the FGF2-NANOG molecular axis as a downstream component of API5 signaling that is conserved in cervical cancer patients, as well as an *in vivo* zebrafish model. Finally, we demonstrate that the blockade of FGFR signaling is an effective strategy to control API5^high^ CSC-like cancer cells.

## Results

### API5 is required for maintenance of CSC-like properties

**Figure 1:**
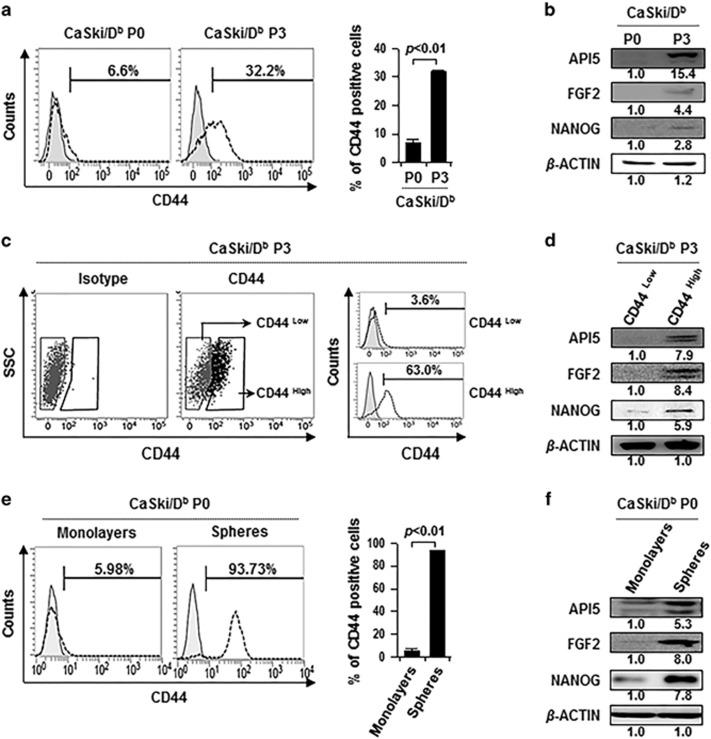
API5 expression is increased in cancer stem-like cell-enriched populations. (**a**) Flow cytometry analysis of CD44 expression in CaSki/D^b^ P0 and P3 cells. The bar graph depicts the percentage of CD44 (mean±s.d.). (**b**) Western blot analysis of expression of API5, FGF2 and NANOG in CaSki/D^b^ P0 and P3 cells. (**c**) CD44^high^ or CD44^low^ cells were sorted from CaSki/D^b^ P3 cells by using FACSAria III flow cytometer (left panel). Flow cytometry analysis of CD44 expression in CD44^high^ or CD44^low^ cells (right panel). (**d**) Western blot analysis of expression of API5, FGF2 and NANOG in CD44^high^ or CD44^low^ cells. (**e**) Flow cytometry analysis of CD44 in CaSki cells grown in monolayer cultures (monolayers) and spheres isolated from CaSki cells under suspension conditions (spheres). The bar graph depicts the percentage of CD44 (mean±s.d.). (**f**) Western blot analysis of expression of API5, FGF2 and NANOG in spheres and monolayers of CaSki cells. (**b**, **d**, **f**) Numbers below blots indicate the expression as measured by fold change.

Previously, we reported that the immune selection-derived CaSki/D^b^ P3 cells (hereafter designated P3), in contrast to their parental counterparts CaSki/D^b^ P0 cells (hereafter designated P0), exhibited CSC-like phenotypes, including enhanced capacities for *in vitro* sphere formation and *in vivo* tumorigenicity, and possessed the CSC surface marker CD44 and stemness factor NANOG.^11^ To examine whether API5 is involved in the maintenance of CSC-like cells, we assessed API5 expression in highly CSC-enriched populations, including the P3 immune selection-derived cell line, CD44^high^ cells and sphere-forming cells, respectively. Compared with P0 cells, P3 cells were five times more enriched in cells expressing CD44, which is a prominent CSC surface marker of CaSki cells,^11^ and had higher levels of API5 and NANOG (Figures 1a and b). As CD44 has been used to enrich putative CSCs in various types of solid cancer and is a prominent CSC surface marker of CaSki cells,^2, 11^ we purified CD44^low^ and CD44^high^ cells from P3 cells by cell sorting with fluorescence-activated cell sorting (FACS). This separation produced >17.5-fold enrichment of CD44 expression (Figure 1c). Notably, CD44^high^ cells expressed higher levels of API5 as compared with CD44^low^ cells, suggesting that expression of API5 co-segregates with CD44 (Figure 1d). It has also been shown that isolation of tumor cells from mammo-spheres can enrich populations of tumor-initiating CSCs.^8, 9^ Based on this rationale, we isolated primary spheres from CaSki cells under suspension conditions. As expected, we observed enrichment of CD44-expressing cells and increased expression of the stemness factor NANOG in spheres isolated from CaSki cells as compared with CaSki cells grown in monolayer cultures (Figures 1e and f). API5 protein expression was significantly increased in spheres, compared with monolayers (Figure 1f). The results, which show that API5 expression is increased in CSC-enriched populations, suggest that API5 may be associated with CSC-like properties.

**Figure 2:**
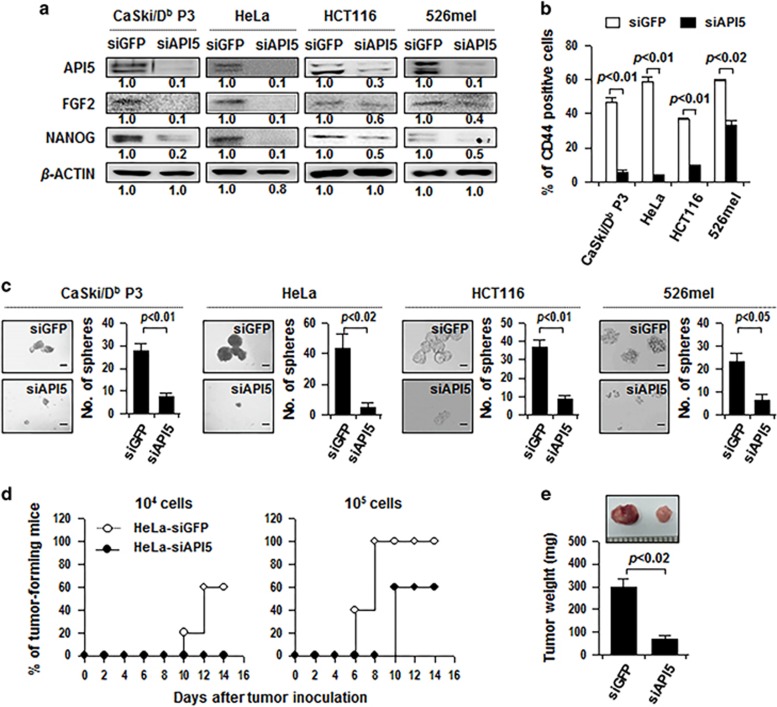
API5 expression is required for CSC-like properties. (**a**–**c**) CaSki/D^b^ P3, HeLa, HCT116 and 526mel cells were transfected with siGFP or siAPI5. (**a**) Western blot analysis of expression of API5, FGF2 and NANOG in these cells. Numbers below blots indicate the expression as measured by fold change. (**b**) Flow cytometry analysis of CD44 in these cells. Error bars represent mean±s.d. Data presented are representative of three independent experiments. (**c**) *In vitro* tumor sphere-forming assay in low-density suspension cultures (500 cells per well). Error bars represent mean±s.d. Data presented are representative of three independent experiments. (**d**) *In vivo* tumorigenicity of HeLa-siGFP cells versus HeLa-siAPI5 cells inoculated at indicated doses into five NOD/SCID mice per group. (**e**) Tumor burden 12 days after injection of 10^5^ cancer cells.

To further assess the role of API5 in the maintenance of CSC-like properties, we silenced API5 by treatment with small interfering RNA (siRNA) in CaSki P3 cells, and HeLa, HCT116, and 526mel cells, which have high levels of API5 expression.^18^ When we silenced API5 in cells, NANOG expression, the frequency of CD44-positive population and sphere-forming capacity were significantly reduced (Figures 2a–c). Moreover, siAPI5-treated HeLa cells had reduced *in vivo* tumorigenicity when transplanted into NOD/SCID mice, compared with siGFP-treated HeLa cells (Figures 2d and e). Taken together, these results indicate that API5 expression is necessary for maintenance of CSC-like properties in multiple human cancer cells.

### API5 promotes CSC-like properties

**Figure 3:**
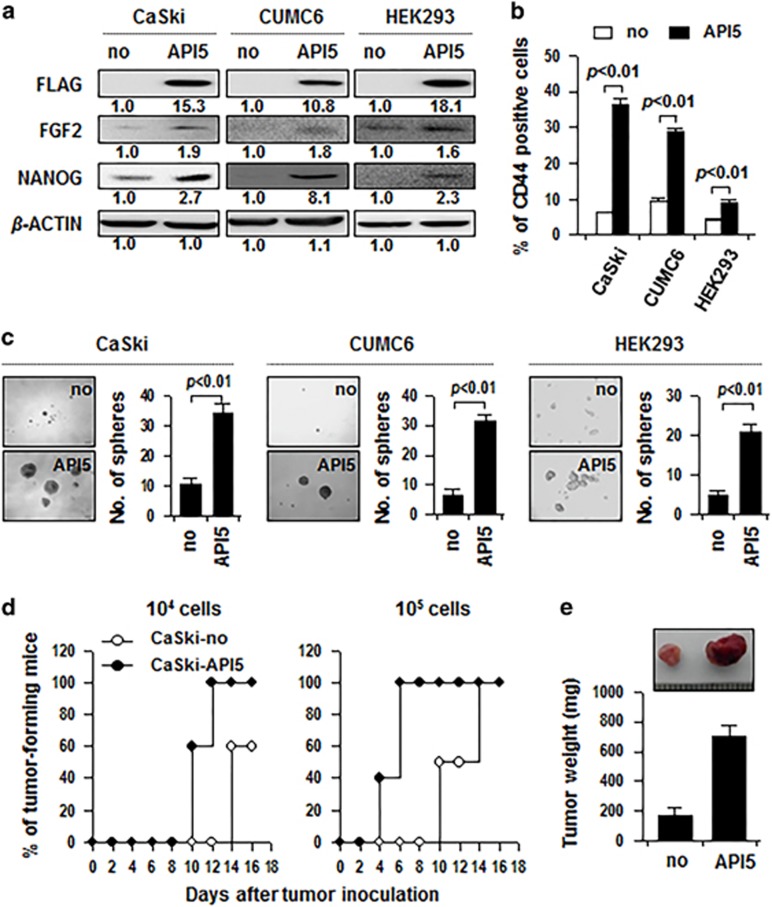
API5 promotes CSC-like properties. (**a**–**c**) CaSki, CUMC6 and HEK293 cells were stably transfected with pMSCV-no insert (no) or pMSCV-API5 (API5). (**a**) Western blot analysis of expression of API5, FGF2 and NANOG in these cells. Numbers below blots indicate the expression as measured by fold change. (**b**) Flow cytometry analysis of CD44 in these cells. Error bars represent mean±s.d. Data presented are representative of three independent experiments. (**c**) *In vitro* tumor sphere-forming assay in low-density suspension cultures (500 cells per well). Error bars represent mean±s.d. Data presented are representative of three independent experiments. (**d**) *In vivo* tumorigenicity of CaSki-no versus CaSki-API5 cells inoculated at indicated doses into five NOD/SCID mice per group. (**e**) Tumor burden 12 days after injection of 10^5^ cancer cells.

To explore whether API5 expression alone can induce CSC-like properties, we tested the possibility that ectopic expression of API5 is indeed sufficient to induce CSC-like properties, both *in vitro* and *in vivo*. API5 overexpression increased NANOG expression, the frequency of CD44-positive cells and sphere-forming capacity in CaSki, CUMC6 and HEK293 cells (Figures 3a–c). Furthermore, API5-overexpressing CaSki (CaSki-API5) cells were more tumorigenic than control cells (CaSki-no) when transplanted into NOD/SCID mice (Figures 3d and e). Actually, as few as 10^4^ CaSki-API5 cells could robustly initiate tumors (10/10 sites), whereas at least 10^5^ CaSki-no cells were required to obtain a 50% tumor take rate (5/10 sites) within 12 days after injection of these cells (Figure 3d). Collectively, these findings indicate that API5 expression by itself is sufficient to promote CSC-like properties.

### FGF2 signaling is crucial for CSC-like properties induced by API5

**Figure 4:**
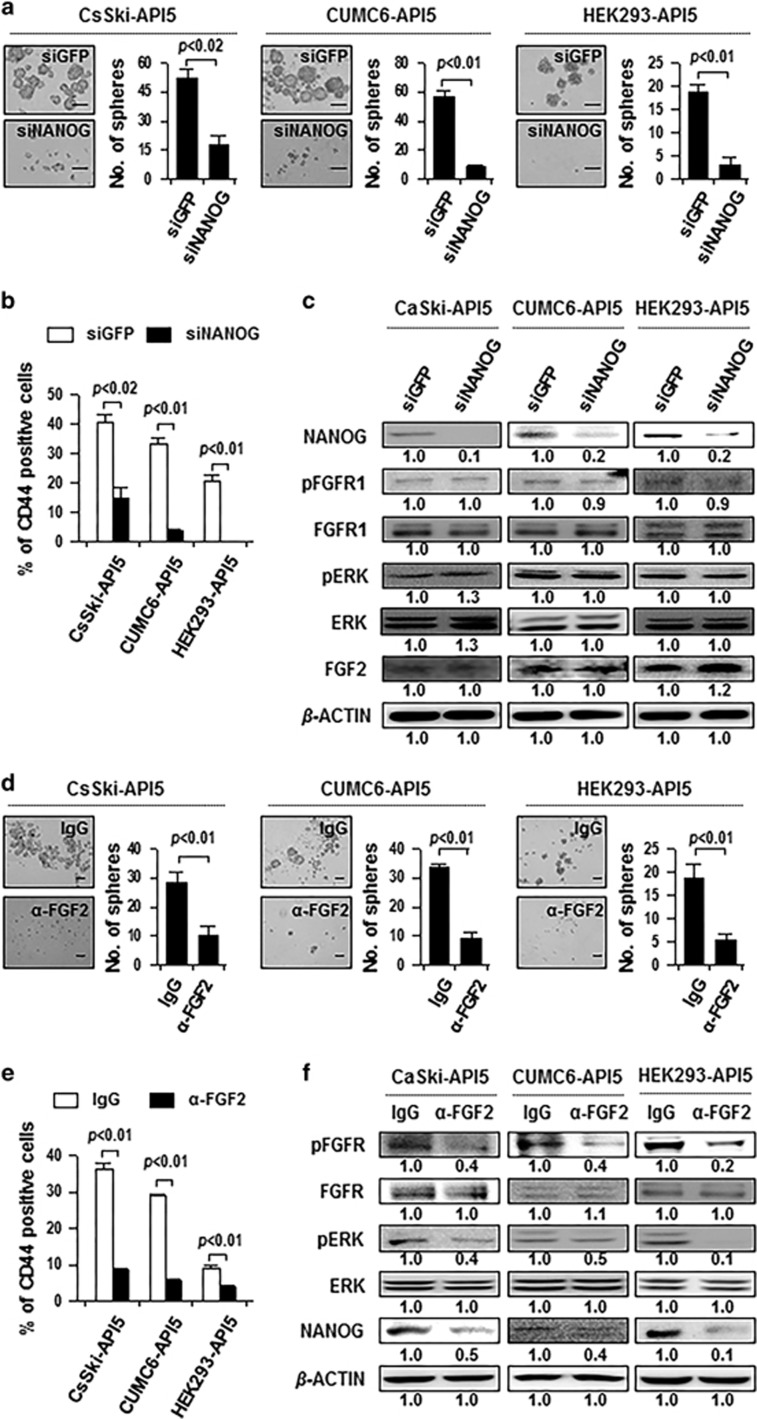
FGF2 signaling is crucial for CSC-like properties induced by API5. (**a**–**c**) CaSki-API5, CUMC6-API5 and HEK293-API5 cells were transfected with siGFP or siNANOG. (**d**–**f**) CaSki-API5, CUMC6-API5 and HEK293-API5 cells were treated with IgG isotype controls (IgG) or anti-FGF2 neutralizing antibody (α-FGF2). (**a**, **d**) *In vitro* tumor sphere-forming assay in low-density suspension cultures (500 cells/well). Error bars represent mean±s.d. Data presented are representative of three independent experiments. (**b**, **e**) Flow cytometry analysis of CD44 in these cells. Error bars represent mean±s.d. Data presented are representative of three independent experiments. (**c**, **f**) Expression levels of NANOG, FGF2, pFGFR, FGFR, pERK and ERK in these cells were analyzed by western blot analysis. Numbers below blots indicate the expression as measured by fold change.

We previously identified NANOG as one of the key molecules that drive the stem-like property of immune-resistant cancer cells.^11, 12^ We wondered whether NANOG is also required for CSC-like properties induced by API5. Indeed, silencing of NANOG in API5-overexpressing CaSki, CUMC6 and HEK293 cells significantly decreased the sphere-forming capacity and frequency of CD44-positive cells (Figures 4a and b), indicating that NANOG has a crucial role in API5-mediated CSC-like property. On the other hand, we previously found that tumor immune resistance conferred by API5 is attributable to the upregulation of FGF2 and the activation of a downstream pathway involving FGFR1/PKCδ/ERK.^18^ In this study, FGF2 expression was increased in all of the above CSC-enriched conditions and it corresponded closely to API5 and NANOG expression (Figure 1). These results strongly suggest that FGF2 signaling could also be critical for API5-mediated CSC properties. To test this possibility, we treated API5-overexpressing cell lines (CaSki-API5, CUMC6-API5 and HEK293-API5) with siRNA targeting FGF2 or green fluorescent protein (GFP) control. We observed that administration of siFGF2 reduced the sphere-forming ability of these API5-overexpressing cells (Supplementary Figure S1). In addition to knockdown of FGF2, neutralization of secretable FGF2 by mAbs (α-FGF2) could effectively block FGFR1 signaling triggered by API5.^18^ Notably, antibody blockade of FGF2 led to a decrease in pFGFR1 and pERK in CaSki-API5, CUMC6-API5 and HEK293-API5 cells (Figure 4f). These intracellular signaling events, occurring in the presence of neutralizing anti-FGF2 Abs, were accompanied by decreased sphere-forming abilities (Figure 4d) and decreased frequency of CD44-positive cells (Figure 4e) in CaSki-API5, CUMC6-API5 and HEK293-API5 cells. These data indicate that both NANOG and FGF2 control the CSC-like properties induced by API5. To clarify the hierarchical relationship between NANOG and FGF2 signaling in API5-mediated CSC-like property, we transfected siRNA targeting NANOG or treated α-FGF2 Ab into API5-overexpressing cells. When we silenced NANOG, FGF2 expression and levels of pFGFR and pERK were unaffected (Figure 4c), although API5-mediated CSC-like properties were efficiently decreased. However, treatment with α-FGF2 significantly decreased NANOG expression, along with blocking of FGFR signaling (Figure 4f), suggesting a direct role of FGF2 in API5-induced NANOG expression. Taken together, these results suggest that API5 confers CSC-like property in cancer cells through the FGF2-NANOG molecular axis.

### API5 regulates FGF2 mRNA expression by modulating E2F1 binding to the FGF2 promoter

**Figure 5:**
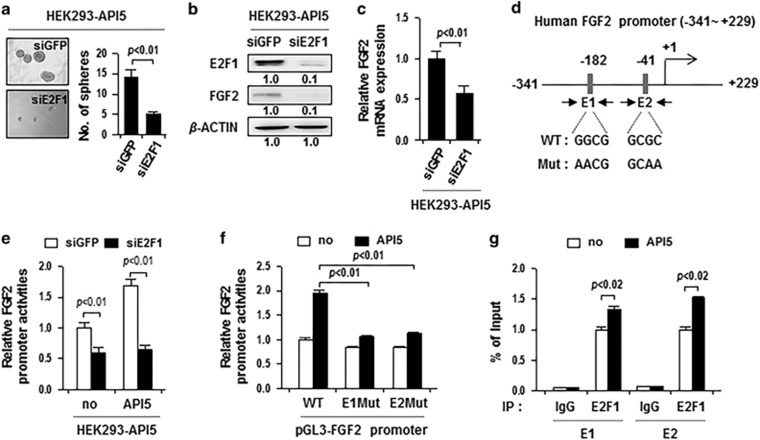
API5 regulates FGF2 mRNA expression by modulating E2F1 binding to the FGF2 promoter. (**a**–**c**) HEK293-no insert and HEK293-API5 cells were transfected with siGFP and siE2F1. (**a**) *In vitro* tumor sphere-forming assay in low-density suspension cultures (500 cells per well). Error bars represent mean±s.d. Data presented are representative of three independent experiments. (**b**) FGF2 protein expression was analyzed by western blot analysis. Numbers below blots indicate the expression as measured by fold change. (**c**) FGF2 mRNA expression was evaluated by real-time pPCR analysis. (**d**) Diagram of the FGF2 promoter region (−341 to +229) containing two E2F1-binding elements. The arrows indicate ChIP amplicon corresponding to E1 and E2. (**e**) Luciferase enzymatic assay in HEK293-no insert and HEK293-API5 cells transfected with the pGL3-FGF2 promoter, together siGFP or siE2F1. (**f**) Luciferase enzymatic assay in HEK293 cells transfected with pGL3-FGF2 WT, E1 Mut or E2 Mut. (**g**) ChIP assay was carried out using HEK293-no insert and HEK293-API5 cells. Cross-linked chromatin was immunoprecipitated with anti-E2F1 antibodies. Immunoprecipitated DNAs were amplified with PCR primers specific for the FGF2 promoter region indicated above. Mouse IgG was used as a negative control. The ChIP data represent IP values for each region's relative ratio to the input.

Although it is certain that FGF2 expression is a critical determinant of CSC properties and immune resistance mediated by API5, the regulatory mechanism for API5-mediated FGF2 expression is still unknown. Our previous report demonstrated that API5 regulates mRNA expression of FGF2 in various cancer cells including A375, HeLa and 526mel.^18^ It was suggested that API5 positively contributes to E2F1-mediated transcriptional activity.^24^ As the report strongly suggests that API5 is functionally related to E2F1, we tested E2F1 dependency in API5-mediated sphere-forming capacity. As a result, knockdown of E2F1 decreased the sphere-forming capacity of HEK293-API5 cells, suggesting that E2F1 is important for API5-mediated CSC property (Figure 5a). We next investigated whether API5-mediated FGF2 expression is dependent on E2F1 expression. Silencing of E2F1 decreased FGF2 expression at both protein and mRNA levels in HEK293-API5 cells (Figures 5b and c). To determine the direct regulation of FGF2 by E2F1, we identified the putative E2F1-binding sites in the 5′-flanking region of the FGF2 gene, and generated a report construct (Figure 5d). Consistent with the change in mRNA level of FGF2, siE2F1 led to a more than twofold decrease in FGF2 promoter activity, compared with siGFP, in HEK293-API5 cells (Figure 5e). Consequently, mutation of E2F1-binding sites in the FGF2 promoter (E1 Mut and E2 Mut) significantly decreased luciferase activity in HEK293-API5 cells but not in HEK293-no cells (Figure 5f). Chromatin immunoprecipitation (ChIP) assay confirmed direct binding of E2F1 to the FGF2 regulatory region (Figure 5g). In addition, API5 results in an increase in E2F1 binding to their binding sites in the FGF2 regulatory region (Figure 5g), as shown in the previous report.^24^ Collectively, these data indicate that API5 overexpression regulates FGF2 mRNA expression by modulating E2F1 binding to the FGF2 promoter.

### Ectopic expression of API5 induces FGF2 and NANOG expression *in vivo*

**Figure 6:**
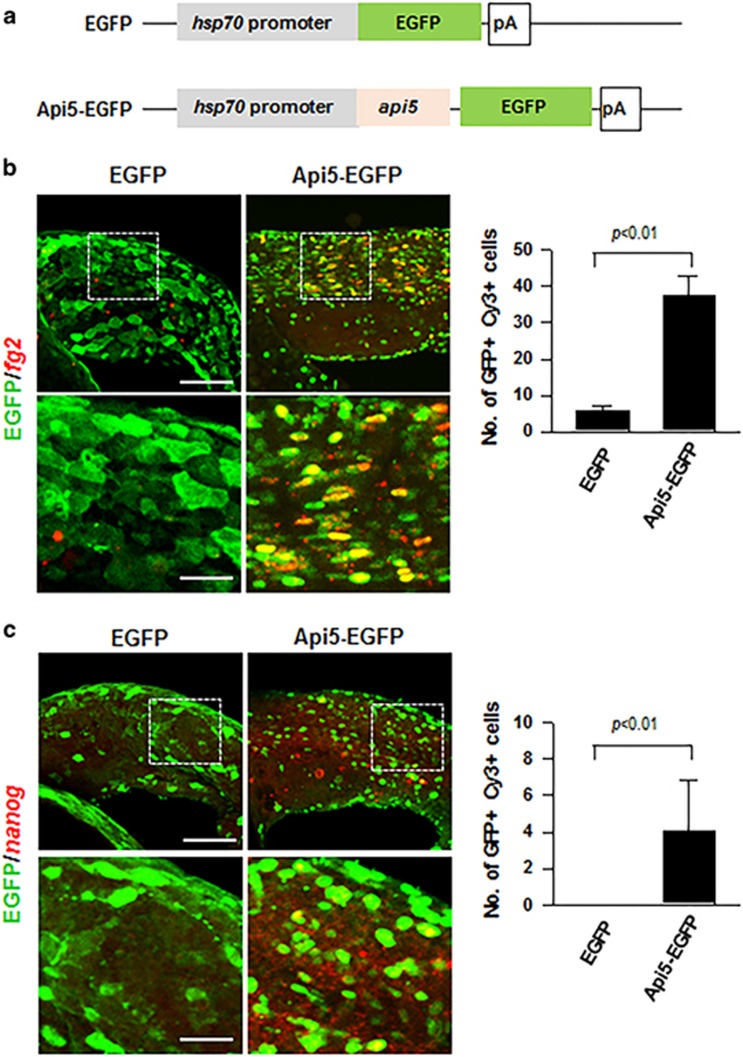
Ectopic expression of *api5* induces *fgf2* and *nanog* expression *in vivo*. (**a**) Structure of the *hsp70*:*egfp* and *hsp70*:*api5*-*egfp* DNA to drive EGFP or API5-EGFP, respectively, under the control of heat-shock 70 promoter. All images are lateral views of the spinal cord of zebrafish embryo with anterior to the left and dorsal to the top. Zebrafish embryos were heat shocked at 22 hpf after injection of *egfp* or *api5-egfp* DNA. All embryos were collected at 24 hpf and labeled by fluorescent *in situ* RNA hybridization with *fgf2* (**b**) and *nanog* (**c**) probes (red color). Lower panels show high magnification images of the boxed areas in upper panels. Graph shows quantification of the number of EGFP^+^Cy3^+^ cells (*P*<0.001, *P*<0.01 each, respectively). Data were obtained from each of the six larvae. Scale bars: upper panel 100 μm, lower panel 20 μm.

We next examined whether ectopic expression of *api5* is sufficient to induce FGF2 and NANOG expression *in vivo* in zebrafish. For ectopic expression of *api5*, we first generated recombinant DNA constructs, which express enhanced green fluorescent protein (EGFP) (*hsp70:egfp*) alone or API5-EGFP fusion protein (*hsp70:api5-egfp*) under the control of heat-shock 70 promoter (Hsp70); (Figure 6a). We next injected recombinant plasmid DNA encoding Hsp70:EGFP or Hsp70:API5-EGFP into one-cell stage wild-type embryos. After heat-shock induction of the injected embryos at 22 hours post-fertilization (hpf), we performed fluorescence *in situ* hybridization using the probes against *fgf2* and *nanog*. In the embryos injected with EGFP alone, we could rarely detect FGF2 expression in the EGFP-expressing cells. However, FGF2 expression is detectable in the majority of API5-EGFP-expressing cells (Figure 6b). The number of FGF2^+^/API5-EGFP^+^ cells was seven times higher than that of FGF2^+^/EGFP^+^ cells. We next tested whether ectopic expression of *api5* can induce *nanog* expression. Previous studies have reported that endogenous *nanog* expression is silenced from the gastrula stage (~10 hpf) during zebrafish development.^25^ Also, we could not detect any *nanog* expression in the neuroectoderm of *egfp*-injected zebrafish (Figure 6c). However, we could observe that *nanog* expression was ectopically induced in the *api5-egfp*-expressing cells of zebrafish (Figure 6c). These data are consistent with the result that API5 regulates FGF2 and NANOG expression *in vitro*. In addition, overexpression of API5-induced expression of other stem cell factors including *klf4*, *sox2* and *oct4* (Supplementary Figure S2). Altogether, these data indicate that overexpression of API5 can induce ectopic expression of FGF2 and NANOG in zebrafish embryos.

### API5-FGF2-NANOG expression in tumor cells is associated with prognosis of cervical cancer

**Figure 7:**
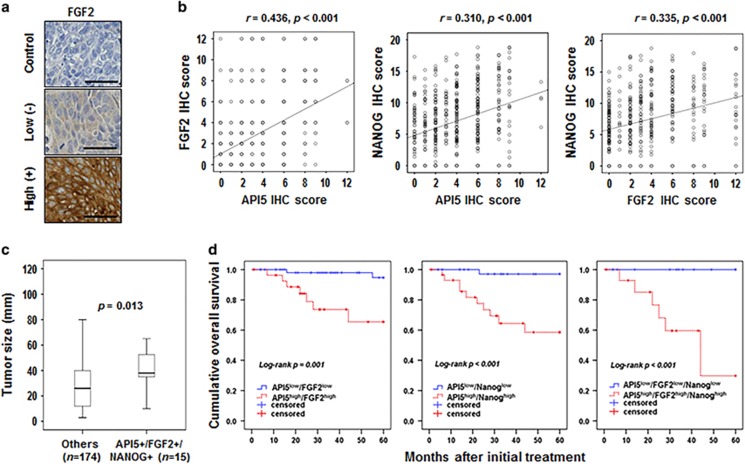
API5, FGF2 and NANOG expression in human cervical neoplasia specimens correlates with the stage and outcome of disease. (**a**) Representative immunohistochemical staining of FGF2 expression in cervical carcinoma specimens (scale bar: 50 μm). (**b**) Correlation between protein expression levels. The expression of API5 was positively correlated with that of FGF2 (Spearman's rho=0.436, *P*<0.001) and NANOG (Spearman's rho=0.310, *P*<0.001). (**c**) API5^+^/FGF2^+^/NANOG^+^ expression was strongly associated with large-sized tumors (*P*=0.013). (**d**) Overall survival curves for cervical cancer patients according to combined marker groups. Patients with combined API5^+^/FGF2^+^, API5^+^/Nanog^+^ and API5^+^/FGF2^+^/NANOG^+^ expression showed significantly worse overall survival (75.0%, 66.7% and 60.0%, respectively) than patients with combined API5^−^/FGF2^-^, API5^−^/NANOG^−^ and API5^−^/FGF2^−^/NANOG^−^ expression (96.7%, 97.8% and 100%, respectively).

We have previously reported that high expression of API5 and NANOG was correlated with poor prognosis of cervical carcinoma.^11, 19^ Here, we evaluated FGF2 expression by immunohistochemistry in the same patient population (Figure 7a), and further analyzed its relationship with API5, FGF2 and NANOG in the development and progression of cervical cancer. FGF2 expression increased during tumor progression from normal to cancer states (*P*<0.01, Supplementary Figure S3a). The correlation between the expression of API5 and that of FGF2 and/or NANOG was assessed in cervical neoplasia patients. The expression of API5 was positively correlated with that of FGF2 (Spearman's rho=0.436, *P*<0.001) and NANOG (Spearman's rho=0.310, *P*<0.001) (Figure 7b). FGF2 expression was also positively correlated with NANOG expression (Spearman's rho=0.335, *P*<0.001) (Figure 7b). Notably, a larger tumor size of cervical neoplasia had a significant correlation with triple-positive API5^+^/FGF2^+^/NANOG^+^ expression (Figure 7c). We next examined the relationship of expression of each protein with patient survival outcomes. Kaplan–Meier plots demonstrated that patients with high FGF2 expression displayed a tendency toward worse 5-year overall survival (86.4% versus 92.5%, *P*=0.170, Supplementary Figure S3b). Furthermore, patients with combined API5^+^/FGF2^+^, API5^+^/NANOG^+^ and API5^+^/FGF2^+^/NANOG^+^ expression showed significantly worse overall survival (75.0% versus 96.7%, *P*=0.001; 66.7% versus 97.8%, *P*<0.001; and 60.0% versus 100%, *P*<0.001, respectively) than patients with API5^−^/FGF2^−^, API5^−^/NANOG^−^ and API5^−^/FGF2^−^/NANOG^−^ expression (Figure 7d). The Cox proportional hazards model revealed that API5^+^/FGF2^+^, API5^+^/NANOG^+^ and API5^+^/FGF2^+^/NANOG^+^ expression levels were independent prognostic factors with respect to overall survival (Supplementary Table S1). Overall, these data indicate that the API5-FGF2-NANOG axis serves as an important prognostic factor in human cervical neoplasia.

### Targeting FGFR signaling reduces API5-mediated CSC-like properties and leads to tumor regression

**Figure 8:**
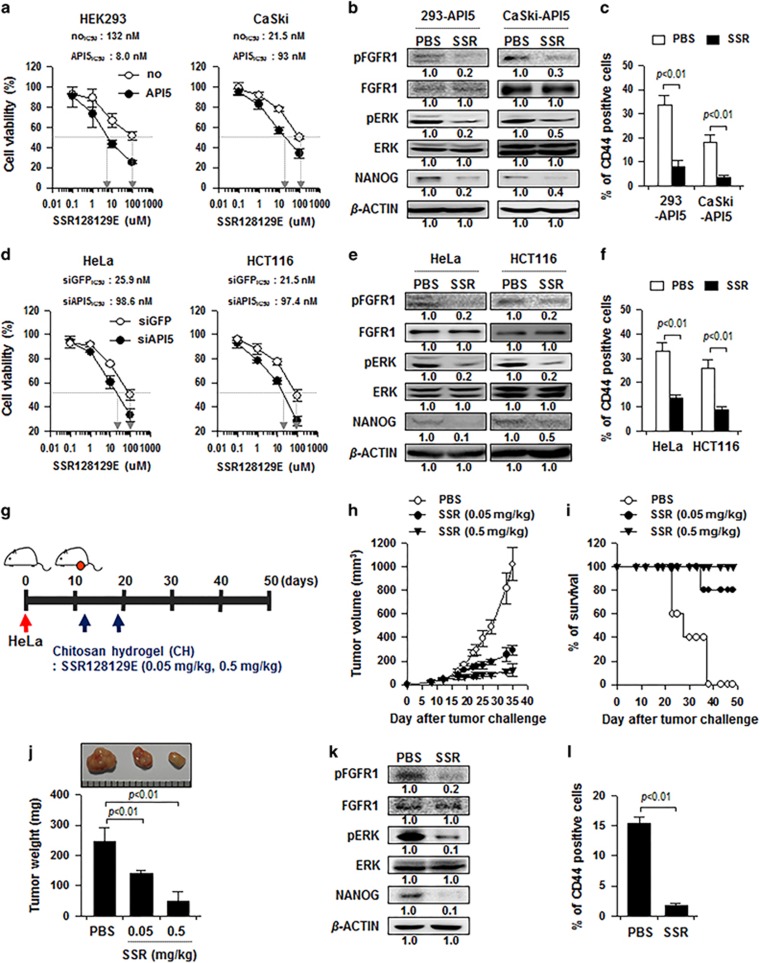
Targeting FGFR signaling reduces API5-mediated CSC-like properties and leads to tumor regression. (**a**, **d**) Cells were treated with indicated concentrations of SSR128129E for 48 h. Cell viability was measured by an MTT assay, and then the concentrations resulting in 50% inhibition of cell viability (IC50 values) were determined. (**b**, **e**) Cells were treated with 10 uM of SSR128129E for 12 h. Expression levels of NANOG, pFGFR, FGFR, pERK and ERK in these cells were analyzed by western blot analysis. Numbers below blots indicate the expression as measured by fold change. (**c**, **f**) Cells were treated with 10 uM of SSR128129E for 12 h. Flow cytometry analysis of CD44 in these cells. Error bars represent mean±s.d. Data presented are representative of three independent experiments. (**g**) Schematic of the therapy regimen in mice implanted with HeLa breast cancer cells. (**h**) Tumor growth and (**i**) survival of mice inoculated with HeLa treated with indicated doses of SSR128129E (5 mice per group). (**j**) Tumor mass of mice at 35 days after challenge. (**k**) Western blot analysis of NANOG, pFGFR, FGFR, pERK and ERK expression in mice administered with PBS or SSR128129E. (**l**) Flow cytometry analysis of the frequency of CD44-positive cells in the tumors of mice.

As FGF2-FGFR1 signaling is critical for API5-mediated phenotypes of cancer cells, we reasoned that inhibition of FGFR1 signaling may serve as an effective strategy for targeting cancer cells, which highly express API5. To evaluate this idea, we chose SSR128129E, which is an allosteric inhibitor of FGF receptor signaling at nanomolar concentrations.^26^ We examined whether API5 is required for susceptibility of cancer cells to SSR128129E. Overexpression of API5 in HEK293 and CaSki cells increased the sensitivity to SSR128129E (Figure 8a). Conversely, silencing of API5 decreased the sensitivity of HeLa and HCT116 cells to SSR128129E (Figure 8d). Notably, treatment with SSR128129E did in fact decrease the levels of pFGFR, pERK and NANOG proteins, as well as the sphere-forming capacity of HEK293-API5, CaSki-API5, HeLa and HCT116 cells (Figures 8b). These results suggest that API5 is a key mediator that determines susceptibility to SSR128129E, and FGFR1 inhibition with SSR128129E is an efficient chemo-therapeutic strategy targeting API5-mediated CSC-like property.

To demonstrate the therapeutic value of FGFR inhibition and its downstream molecular axis *in vivo*, the efficacy of SSR128129E was tested in nude mice bearing HeLa tumors. As described in Figure 8g, 12 days after tumor challenge, mice were intra-tumorally injected with two different doses (0.05 and 0.5 mg/kg) of SSR128129E. Tumors excised on day 35 were substantially smaller in size and weight among mice that received SSR128129E compared with mice that received phosphate-buffered saline (PBS; Figures 8h and j). Importantly, 100% of mice that received 0.5 mg/kg of SSR128129E survived, even 48 days after tumor challenge; in contrast, all of the animals in the control group had died by that time (Figure 8i). Western blot analysis of *ex vivo* isolated tumors at day 35 after challenge demonstrated decreased levels of pFGFR, pERK and NANOG proteins among mice that received 0.5 mg/kg of SSR128129E (Figure 8k), demonstrating achievement of successful *in vivo* delivery of SSR128129E to the tumor. In addition, the SSR128129E-treated tumors contained fewer CD44-positive cells than the PBS-treated tumors (Figure 8l). Taken together, our data show that inhibition of FGFR represents an attractive strategy for the control of API5^high^ human cancer.

## Discussion

CSCs have a prominent role in the maintenance and progression of certain cancers. We previously demonstrated that immune pressure enforced through vaccination drives the evolution of tumor cells toward a phenotype resembling CSCs.^11, 12^ We also found that API5 acts as a novel immune-resistant factor, which confers anti-apoptotic property to cancer cells by activating FGF2 signaling.^18^ Although recent studies suggest that API5 has oncogenic potential in numerous human cancers in addition to its primary role for anti-apoptotic properties, its functional association with CSC-like properties remains largely unknown. Here, we found that API5 mediates CSC-like properties through E2F1-dependent FGF2 expression, resulting in activation of the FGFR1-ERK signaling pathway.

Our previous study demonstrated that NANOG, a transcription factor pivotal in self-renewal of embryonic stem cells, is sufficient to enhance the CSC-like and immune-resistant phenotype during the immune selection process.^11, 12^ However, it remains largely unknown what factors potentiate NANOG expression in immune-resistant cancer cells. In this study, we report that API5 is capable of regulating NANOG expression by activating FGF2 signaling, thereby promoting CSC-like phenotypes. Notably, after silencing of NANOG, the CSC-like phenotypes of API5-overexpressing cells were almost entirely lost, indicating that NANOG is a key downstream component involved in API5-mediated CSC-like property. Moreover, despite API5 overexpression, blockade of FGF2 signaling using various methods including FGF2 targeted siRNA, FGF2 neutralizing Abs and chemical inhibitor of FGFR, leads to decrease in NANOG expression, as well as CSC-like property. These data indicate that the FGF2 signaling pathway is the primary route through which API5 acts to regulate NANOG expression and then to promote the phenotype. Collectively, besides its ability to confer anti-apoptotic property to cancer cells, API5 is also able to turn tumor cells into CSC-like cells by regulating NANOG expression.

Previously, we demonstrated that expression of API5 and NANOG in tumor tissue correlated with stage and outcome of disease in patients with cervical neoplasia.^11, 19^ In this study, we found that FGF2 expression increased as tumors progressed from normal to cancer states. Importantly, FGF2 expression was positively correlated with expression of API5 and NANOG in cervical cancer tissue, validating the biochemical pathway that we proposed. We presented the supporting data in this study, which shows that the API5-FGF2-NANOG molecular axis may be critically related to progression of cervical neoplasia, and the expression level of the molecular axis component is correlated with the disease stage or prognosis degree in patients.

It is certain that induction of FGF2 expression is critical for triggering the FGFR1-ERK signaling pathway in API5-mediated CSC-like phenotypes. Here, we further elucidated the molecular mechanism by which API5 regulates FGF2 expression. Previous reports emphasized that API5 function is closely related to the E2F1 transcription factor.^16, 24^ Our data also show that E2F1 may participate in API5-mediated CSC-like property, because the knockdown of E2F1 in API5-expressing cells resulted in a decrease in the sphere-forming capacity, as well as the expression of FGF2 and NANOG. A previous report proposed that *Drosophila* API5 suppresses E2F1-dependent apoptosis without generally altering E2F1-dependent transcription.^16^ Meanwhile, a contrary observation, suggesting that API5 contributes to E2F1-dependent transcriptional activation of cell cycle-associated genes by facilitating E2F1 recruitment onto its target promoters, was also reported.^24^ Along with conclusions of latter report, our data showed that API5 upregulates FGF2 expression by modulating E2F1 binding to the FGF2 promoter. However, precise underlying mechanism(s) by which API5 regulates E2F1 transcriptional activity needs further study.

As FGF2-FGFR signaling has been implicated as a central channel in the development of CSC-like phenotypes by API5, we believe that inhibition of FGFR signaling may be an effective strategy to control human cancer. Currently, a number of chemical inhibitors against FGFR have been developed, considering the important biological impact of FGFs and their receptors in tumor cells. Among them, SSR128129E is an orally active and allosteric FGFR inhibitor that has no effects on the other types of receptor tyrosine kinases.^27^ In this study, to evaluate the therapeutic potential of FGFR inhibition and its downstream molecular axis *in vivo*, we tested the efficacy of SSR128129E by intra-tumoral injection of chitosan hydrogel containing SSR128129E into mice harboring high API5-expressing HeLa cells. Indeed, SSR128129E treatment led to significant antitumor effects along with prolongation of survival in mice. Moreover, the antitumor effect of SSR128129E may be accompanied by suppression of CSC-like property, as evidenced by decreased population of CD44-positive cells in SSR128129E-treated tumors compared with PBS-treated tumors.

In conclusion, we demonstrated that API5 confers CSC-like property by upregulating NANOG expression. Critically, these CSC-like properties mediated by API5 are dependent on FGFR1 signaling, which is triggered by E2F1-dependent FGF2 expression. Furthermore, we uncovered the FGF2-NANOG molecular axis as a downstream component of API5 signaling that is conserved in cervical cancer patients, as well as an *in vivo* zebrafish model. Finally, our findings propose that the blockade of FGFR signaling may be a promising therapeutic approach for cancer, especially if there is high API5 expression.

## Materials and methods

### Mice

Six- to eight-week-old female NOD/SCID and nude mice were purchased from Central Lab. Animal Inc. (Seoul, Korea). All mice were maintained and handled under the protocol approved by the Korea University Institutional Animal Care and Use Committee (KUIACUC-2009-126). All animal procedures were performed in accordance with recommendations for the proper use and care of laboratory animals.

### DNA constructs

The pMSCV-hAPI5 plasmids have been previously described.^18^ To generate the pGL3-FGF2 promoter, the promoter region of the FGF2 gene was isolated by PCR from genomic DNA extracted from CaSki cells using the primer set, 5′-AACTCGAGTGGGGTGGAAACGGCTTCTC-3′ (forward) and 5′-AGAAGCTTTTCACGGATGGGTGTCTCCG-3′ (reverse). The PCR products were digested with *Xho*I and *Hind*III and subcloned into the *Xho*I/*Hind*III restriction sites of the pGL3-Basic vector (Promega, Madison, WI, USA). To construct *hsp70:egfp* and *hsp70:api5-egfp* recombinant DNA, we first amplified zebrafish *api5* using a forward primer containing an attB1 site (5′-GGGGACAAGTTTGTACAAAAAAGCAGGCTGATGGCGGCGACAGTGGAGG-3′) and a reverse primer containing an attB2 site (5′-GGGGACCACTTTGTACAAGAAAGCTGGGTAATAAATCCGGCCTCGGCTTC-3′). The PCR product was cloned into pDONR 221 using BP clonase of the Gateway system (Invitrogen, Gaithersburg, MD, USA). A 5′ entry clone containing a fragment of the zebrafish *hsp70* promoter and a 3′ entry clone containing the *egfp* and Tol2 destination vector were provided by Chi-Bin Chien.^28^ The Multisite Gateway LR reactions were performed using LR II clonase (Invitrogen) according to the manufacturer's recommendations.

### Site-directed mutagenesis

Site-directed mutagenesis was performed using a QuickChange XL Site-directed Mutagenesis kit (Stratagene, San Diego, CA, USA) according to the manufacturer's instructions. To create mutations in the E2F1-binding sites of the FGF2 promoter region, the following primer sets were used: for pGL3-FGF2 promoter-E1 Mut, 5′-GGTGCGGGGGTTGAACGGGGGTGACTTTTGG-3′ (sense) and 5′-CCAAAAGTCACCCCCGTTCAACCCCCGCACC-3′ (antisense); for pGL3-FGF2 promoter-E2 Mut, 5′-CCGGACTGATGTCGCAAGCTTGCGTGTTGTGG-3′ (sense) and 5′-CCACAACACGCAAGCTTGCGACATCAGTCCGG-3′ (antisense). Mutations were verified by DNA sequencing.

### Cells, cell culture and generation of cell lines

The production and maintenance of CaSki/D^b^ P0 and CaSki/D^b^ P3 cells has been previously described.^29^ The human cancer cell lines, CaSki, CUMC6, HeLa and HCT116, and human embryonic kidney 293 (HEK293) cells were purchased from American Type Culture Collection (ATCC, Rockville, MD, USA). Human melanoma cell line 526mel was kindly provided by Dr M Lotze. HEK293, CUMC6, HeLa and HCT116 cells were cultured in Dulbecco's modified Eagle's medium (DMEM; Thermo Scientific, Waltham, MA, USA) containing 100 units/ml penicillin–streptomycin and 10% fetal bovine serum. CaSki and 526mel cells were cultured in RPMI1640 containing 100 units/ml penicillin–streptomycin and 10% fetal bovine serum. All cells were grown at 37 °C in 5% CO_2_ incubator/humidified chamber. CaSki-no insert, CaSki-API5, CUMC6-no insert, CUMC6-API5, HEK293-no insert and HEK293-API5 cell lines were generated using the constructed pMSCV-no insert or pMSCV-API5 plasmid DNAs. Briefly, pMSCV-no insert or pMSCV-API5 plasmid DNAs were first transfected along with viral packaging plasmids (VSVG and Gag-pol) into HEK293FT cells. Three days after transfection, viral supernatant was filtered through a 0.45 μm filter and infected into CaSki cells, as described previously.^10^ Infected cells were then selected with 1 ug/ml puromycin.

### siRNA constructs

Synthetic siRNA specific for GFP, API5, FGF2 and E2F1 were purchased from Bioneer (Daejeon, Korea); nonspecific GFP, 5′-GCAUCAAGGUGAACUUCAA-3′ (sense), 5′-UUGAAGUUCACCUUGAUGC-3′ (antisense); API5, 5′-GGGUUGUUCAGCCAAAUACUU -3′ (sense), 5′-AAGUAUUUGGCUGAACAACCC-3′ (antisense); FGF2, 5′-GAGAGAGGAGUUGUGUCUA-3′ (sense), 5′-UAGUCACAACUCCUCUCUC-3′ (antisense); E2F1, 5′-ACGCUAUGAGACCUCACUG-3′ (sense), 5′-CAGUGAGGUCUCAUAGCGU-3′ (antisense). siRNA was delivered into six-well plates at a dose of 100 pmol per well using Lipofectamine 2000 (Invitrogen).

### Sphere culture and tumor sphere-forming assay

For gathering cells from spheres, CaSki cells were resuspended in serum-free DMEM-F12 (Thermo Scientific) supplemented with epidermal growth factor (EGF, 20 ng/ml), basic fibroblast growth factor (20 ng/ml) and 1 × B27. Spheres were collected after 7 days, and protein was extracted for WB analysis or dissociated with Accutase (Sigma, St Louis, MO, USA) for FACS analysis. To estimate sphere-forming capacity, cells were plated at 500 cells per well in 12-well, super-low adherence vessels (Corning, Lowell, MA, USA) containing serum-free DMEM-F12 (Thermo Scientific) supplemented with EGF (20 ng/ml) and 1 × B27. Medium was replaced every 3 days to replenish nutrients. Colonies >50 μm in diameter were counted under a microscope.

### *In vivo* tumorigenicity assay

Cells were harvested by trypsin treatment and then washed and resuspended in Opti-MEM. NOD/SCID mice were subcutaneously injected with 10^4^ or 10^5^ cells. Tumor formation was monitored every 2 days. After 14–16 days, tumor tissue was excised and weighed.

### Flow cytometry analysis

First, 1 × 10^5^ cells were dissociated by exposure to accutase solution (Sigma Aldrich, St Louis, MO, USA), washed and resuspended in PBS. The gathered cells were then reacted with fluorescein isothiocyanate-conjugated CD44 (1:400; Miltenyi Biotech, Auburn, CA, USA) or isotype control antibody for 1 h at 4 °C. To isolate CD44^high^ or CD44^low^ cells, stained cells were washed twice, resuspended in PBS and sorted by using FACSAria III flow cytometer (BD Biosciences, San Jose, CA, USA). To detect CD44 expression, stained cells were washed twice and analyzed using a FACSVerse flow cytometer (BD Biosciences). Data acquisition was performed using a FACS Calibur flow cytometer (BD Biosciences) with CellQuest Pro software (BD Biosciences).

### Real-time quantitative RT-PCR

Total RNA was isolated using RNeasy Micro kit (Qiagen, Valencia, CA, USA), and the complementary DNAs were synthesized by reverse transcriptase (RT) using iScript cDNA synthesis kit (Bio-Rad, Hercules, CA, USA), according to the manufacturer's recommended protocol. Real-time quantitative PCR was performed using iQ SYBR Green super mix (Bio-Rad) with the following specific primers: hFGF2 5′-GGCTATGAAGGAAGATGGAAGATT-3′ (forward) and 5′-TGCCACATACCAACTGGTGTATTT-3′ (reverse); β-ACTIN, 5′-CGACAGGATGCAGAAGGAGA-3′ (forward) and 5′-TAGAAGCATTTGCGGTGGAC-3′ (reverse) on a CFX96 real-time PCR detection system. All real-time quantitative PCR experiments were performed in triplicate and quantification cycle (Cq) values were determined using Bio-Rad CFX96 Manager 3.0 software. Relative quantification of the mRNA levels was performed using the comparative Ct method with β-actin as the reference gene.

### Western blot analysis

Lysate extracted from a total of 1 × 10^5^ cells was used to perform western blot analysis. Primary antibodies against pFGFR (Y653/654), FGFR, pERK (T202/Y204) and ERK were purchased from Cell Signaling (Danvers, MA, USA) and used at a 1:5000 dilution. Antibodies against NANOG (1:1000, Abnova, Taipei City, Taiwan); API5, FGF2, E2F1 (1:1000, Santa Cruz Biotechnology, Santa Cruz, CA, USA); β-ACTIN (1:10 000, MBL, Nagoya, Japan) were used for western blotting, followed by the appropriate secondary antibodies conjugated with horseradish peroxidase. Immunoreactive bands were developed with the chemiluminescence ECL detection system (Elpis Biotech, Daejeon, Korea), and signals were detected using a luminescent image analyzer (LAS-4000 Mini, Fujifilm, Tokyo, Japan). *β*-ACTIN was included as an internal loading control. The intensity of the western blot signals was quantified using Multi-gauge software (Fujifilm).

### Luciferase assay

For luciferase assay, cells were maintained in DMEM with 10% fetal bovine serum and seeded at 1 × 10^5^ cells per well in 12-well plates 1 day before the assay. Using Lipofectamine 2000 (Invitrogen), cells were administered with 100 ng of pGL3-FGF2 promoter WT, E1 Mut or E2 Mut, together with 20 ng of CMV/*β*-galactosidase plasmid to normalize transfection efficiency. After 24 h, cells were washed with PBS and permeabilized with Cell Culture Lysis Reagent (Promega). Luciferase activity was measured with a Turner Biosystems TD-20/20 luminometer (Turner BioSystems, Sunnyvale, CA, USA) after addition of 40 μl luciferase assay reagent (Promega). *β*-Galactosidase activity was measured with a uQuant microplate reader (BioTek, Winooski, VT, USA) at 570 nm wavelength after addition of *β*-galactosidase assay reagent containing 1 mM chlorophenol red *β*-d-galactopyranoside substrate (Roche, Mannheim, Germany).

### ChIP assay

The ChIP kit (Millipore, Billerica, MA, USA) was used according to the manufacturer's instructions. Briefly, HEK293-no and HEK293-API5 cells (1 × 10^7^ per assay) were bathed in 1% formaldehyde at 25 °C for 10 min for cross-linking of proteins and DNA and then lysed in sodium dodecyl sulfate buffer containing a protease inhibitor. DNA was sheared into 0.2–1- kb fragments by sonication using a Sonic Dismembrator Model 500 (Fisher Scientific, Pittsburgh, PA, USA). Immunoprecipitation was carried out by incubation with 1 μg of anti-E2F1 or mouse IgG (Upstate Biotechnology, Lake Placid, NY, USA) for 16 h. To reverse the protein-DNA crosslinks, the immunoprecipitated sample and input were incubated at 65 °C overnight. After reversal of cross-linking, DNA fragments were purified on spin columns (Upstate Biotechnology). The region flanking the E2F1-binding sites in the FGF2 promoter region was amplified and quantified from immunoprecipitated chromatin with the following primers: E1, 5′-AAGTTGAGTCACGGCTGGTT-3′ (forward) and 5′-AGGGCTTTGGCATTCCCTG-3′ (reverse), E2, 5′-ACTGATGTCGCGCGCTTG-3′ (forward) and 5′-GTTCTCCTCCCTCCTGCG-3′ (reverse) by real-time qPCR using iQ SYBR Green super mix (Bio-Rad) with CFX96 real-time PCR detection system as described above.

### Zebrafish injection and heat-shock induction

Wild-type AB zebrafish was maintained using standard procedures^30^ and was used in this study. Zebrafish embryos were staged according to hpf as per standard criteria.^31^ For ectopic induction of API5, 20–30 pg of *hsp70:egfp* and *hsp70:api5-egfp* DNA constructs was injected into 1- to 2-cell stage zebrafish embryos. To induce the expression of EGFP alone and API5-EGFP, we raised the injected embryos at 28.5 °C, transferred them to embryo medium (EM) at 39 °C for 30 min at 22 hpf, and continued incubation at 28.5 °C. Then, we screened for EGFP fluorescence and fixed the embryos at 24 hpf.

### Fluorescent *in situ* RNA hybridization

To make an antisense probe, we designed a PCR primer (*fgf2* F: 5′-CAGAGACCGACAGACTTAGGG-3′, *fgf2* R: 5′-GCATTCCTCACAGTCAGACG-3′, *nanog* F: 5′-ATGGCGGACTGGAAGATGCC-3′, *nanog* R: 5′-ACAGCAAAGTTATTCCTTTAGTTGCC-3′, *klf4* F: 5′-ATGGCTCTTGCAGATGCG-3′, *klf4* R: 5′-ACATGTGCCTCTTCATGTGCAG-3′, *sox2* F: 5′-ATGTATAACATGATGGAAACCGAGCTG-3′, *sox2* R: 5′-CATATGCGATAAGGGAATCGTGCCG-3′, *oct4* F: 5′-ATGACGGAGAGAGCGCAGAGC-3′, *oct4* R: 5′-AGCTGGTGAGATGACCCACCAA-3′) and amplified a product from 1-day post-fertilization complementary DNA. The 24 hpf embryos were fixed using 4% paraformaldehyde overnight. Fluorescent *in situ* RNA hybridization was performed using TSA PLUS Cy3 kit (Perkin-Elmer, Wellesley, MA, USA) as previously described.^32^ Fluorescence images were collected using a Zeiss LSM 510 laser scanning confocal microscope (Carl Zeiss, Jena, Germany). We quantified the number of EGFP^+^Cy3^+^ cells throughout the whole zebrafish trunk. Statistical significance was tested with a nonpaired Student's *t*-test.

### Tissue samples and immunohistochemistry

Tissue microarrays containing four 1.0 mm cores from 479 formalin-fixed, paraffin-embedded cervical neoplasia tissue specimens and matched nonadjacent normal cervical epithelial tissue specimens, have been previously described. Tissue specimens were prospectively collected from patients who were admitted to Gangnam Severance Hospital between 1996 and 2010. Some of the paraffin blocks were provided by the Korea Gynecologic Cancer Bank through the Bio & Medical Technology Development Program of the Ministry of Education, Science and Technology, Korea (NRF-2012M3A9B8021800). Tissue samples were collected from patients who provided informed consent. This study was approved by the Institutional Review Board of Gangnam Severance Hospital (Seoul, South Korea) and it was additionally approved by the Office of Human Subjects Research at the National Institutes of Health.

The tissue microarray sections were deparaffinized with xylene and dehydrated through a graded ethanol series. Antigen recovery was performed in heat-activated antigen retrieval pH 9.0 (Dako, Carpinteria, CA, USA). Endogenous peroxidase activity was quenched with 3% H_2_O_2_ in water for 10 min. Nonspecific staining was minimized with a protein block (Dako) for 15 min. The sections were incubated for 2 h with rabbit polyclonal anti-FGF2 antibodies (Santa Cruz, Dallas, TX, USA) at a 1:500 dilution, followed by a standard ABC protocol using EnVision^+^ Dual Link System-HRP (Dako). The reactions was visualized by addition of 3,3-diaminobenzidine substrate for 10 min and lightly counter stained with hematoxylin. The FGF2 staining results were scored based on (a) intensity (categorized as 0 (absent), 1 (weak), 2 (moderate) or 3 (strong)) and (b) the percentage of positively stained epithelial cells (scored as 0 (0–5% positive), 1 (6–25%), 2 (26–50%), 3 (51–75%) or 4 (>75%)). A histoscore was generated by multiplying the mean intensity and percent scores (overall score range, 0–12). NANOG and API5 staining patterns were previously evaluated in the same cohort.

### Tumor treatment experiments

On the day of tumor challenge, tumor cells were harvested by trypsinization, washed once with Opti-MEM (Gibco BRL, Invitrogen, CA, USA) and resuspended in Opti-MEM to the desired concentration for subcutaneous injection. Nude mice were inoculated subcutaneously with 2 × 10^6^ HeLa cells per mouse. Twelve and 19 days following tumor challenge, thermosensitive chitosan hydrogel^29^ containing 0.05 mg/kg of SSR128129E, 0.5 mg/kg of SSR128129E (Selleckchem, Houston, TX, USA) or DMSO was injected intra-tumorally. Mice were monitored for tumor burden and survival for 35 and 50 days after challenge, respectively.

### Statistical analysis

All data are representative of at least three separate experiments. Individual data points were compared by two-tailed Student's *t*-test. For IHC data, statistical analysis was performed using the SPSS version 21 (SPSS Inc., Chicago, IL, USA). The Mann–Whitney *U-*test was used to compare the protein expression levels between each group. The × 2-test was used to assess the associations between molecular markers and tumor size. Receiver operating characteristic curve was used to determine FGF2 as a discriminator between patients with a good and poor prognosis over a range of cut-off points. Analysis of the Spearman rho coefficient was used to assess the correlations between molecular markers. Survival distributions were estimated using the Kaplan–Meier method with the log-rank test. A Cox proportional hazards model was created to identify independent predictors of survival. A value of *P*<0.05 was considered statistically significant.

## References

[bib1] Dalerba P, Cho RW, Clarke MF. Cancer stem cells: models and concepts. Annu Rev *Med* 2007; 58: 267–284. 17002552 10.1146/annurev.med.58.062105.204854

[bib2] Zhou BB, Zhang H, Damelin M, Geles KG, Grindley JC, Dirks PB. Tumour-initiating cells: challenges and opportunities for anticancer drug discovery. Nat Rev Drug Discov 2009; 8: 806–823. 19794444 10.1038/nrd2137

[bib3] Clevers H. The cancer stem cell: premises, promises and challenges. Nat Med 2011; 17: 313–319. 21386835 10.1038/nm.2304

[bib4] Dalerba P, Dylla SJ, Park IK, Liu R, Wang X, Cho RW et al. Phenotypic characterization of human colorectal cancer stem cells. Proc Natl Acad Sci USA 2007; 104: 10158–10163. 17548814 10.1073/pnas.0703478104 PMC1891215

[bib5] Lapidot T, Sirard C, Vormoor J, Murdoch B, Hoang T, Caceres-Cortes J et al. A cell initiating human acute myeloid leukaemia after transplantation into SCID mice. Nature 1994; 367: 645–648. 7509044 10.1038/367645a0

[bib6] Boumahdi S, Driessens G, Lapouge G, Rorive S, Nassar D, Le Mercier M et al. SOX2 controls tumour initiation and cancer stem-cell functions in squamous-cell carcinoma. Nature 2014; 511: 246–250. 24909994 10.1038/nature13305

[bib7] Chiou SH, Wang ML, Chou YT, Chen CJ, Hong CF, Hsieh WJ et al. Coexpression of Oct4 and Nanog enhances malignancy in lung adenocarcinoma by inducing cancer stem cell-like properties and epithelial-mesenchymal transdifferentiation. Cancer Res 2010; 70: 10433–10444. 21159654 10.1158/0008-5472.CAN-10-2638

[bib8] Al-Hajj M, Wicha MS, Benito-Hernandez A, Morrison SJ, Clarke MF. Prospective identification of tumorigenic breast cancer cells. Proc Natl Acad Sci USA 2003; 100: 3983–3988. 12629218 10.1073/pnas.0530291100 PMC153034

[bib9] Singh SK, Clarke ID, Terasaki M, Bonn VE, Hawkins C, Squire J et al. Identification of a cancer stem cell in human brain tumors. Cancer Res 2003; 63: 5821–5828. 14522905

[bib10] Noh KH, Kang TH, Kim JH, Pai SI, Lin KY, Hung CF et al. Activation of Akt as a mechanism for tumor immune evasion. Mol Ther 2009; 17: 439–447. 19107122 10.1038/mt.2008.255 PMC2835083

[bib11] Noh KH, Kim BW, Song KH, Cho H, Lee YH, Kim JH et al. Nanog signaling in cancer promotes stem-like phenotype and immune evasion. J Clin Invest 2012; 122: 4077–4093. 23093782 10.1172/JCI64057 PMC3484451

[bib12] Noh KH, Lee YH, Jeon JH, Kang TH, Mao CP, Wu TC et al. Cancer vaccination drives Nanog-dependent evolution of tumor cells toward an immune-resistant and stem-like phenotype. Cancer Res 2012; 72: 1717–1727. 22337995 10.1158/0008-5472.CAN-11-3758 PMC3319841

[bib13] Mao CP, Wu T, Song KH, Kim TW. Immune-mediated tumor evolution: nanog links the emergence of a stem like cancer cell state and immune evasion. Oncoimmunology 2014; 3: e947871. 25610734 10.4161/21624011.2014.947871 PMC4292413

[bib14] Tewari M, Yu M, Ross B, Dean C, Giordano A, Rubin R. AAC-11, a novel cDNA that inhibits apoptosis after growth factor withdrawal. Cancer Res 1997; 57: 4063–4069. 9307294

[bib15] Van den Berghe L, Laurell H, Huez I, Zanibellato C, Prats H, Bugler B. FIF [fibroblast growth factor-2 (FGF-2)-interacting-factor], a nuclear putatively antiapoptotic factor, interacts specifically with FGF-2. Mol Endocrinol (Baltimore, MD) 2000; 14: 1709–1724. 10.1210/mend.14.11.0556 11075807

[bib16] Morris EJ, Michaud WA, Ji JY, Moon NS, Rocco JW, Dyson NJ. Functional identification of Api5 as a suppressor of E2F-dependent apoptosis *in vivo*. PLoS Genet 2006; 2: e196. 17112319 10.1371/journal.pgen.0020196 PMC1636698

[bib17] Rigou P, Piddubnyak V, Faye A, Rain JC, Michel L, Calvo F et al. The antiapoptotic protein AAC-11 interacts with and regulates Acinus-mediated DNA fragmentation. EMBO J 2009; 28: 1576–1588. 19387494 10.1038/emboj.2009.106 PMC2693147

[bib18] Noh KH, Kim SH, Kim JH, Song KH, Lee YH, Kang TH et al. API5 confers tumoral immune escape through FGF2-dependent cell survival pathway. Cancer Res 2014; 74: 3556–3566. 24769442 10.1158/0008-5472.CAN-13-3225 PMC4394897

[bib19] Cho H, Chung JY, Song KH, Noh KH, Kim BW, Chung EJ et al. Apoptosis inhibitor-5 overexpression is associated with tumor progression and poor prognosis in patients with cervical cancer. BMC Cancer 2014; 14: 545. 25070070 10.1186/1471-2407-14-545 PMC4125689

[bib20] Krejci P, Pejchalova K, Rosenbloom BE, Rosenfelt FP, Tran EL, Laurell H et al. The antiapoptotic protein Api5 and its partner, high molecular weight FGF2, are up-regulated in B cell chronic lymphoid leukemia. J Leukocyte Biol 2007; 82: 1363–1364. 17827341 10.1189/jlb.0607425

[bib21] Sasaki H, Moriyama S, Yukiue H, Kobayashi Y, Nakashima Y, Kaji M et al. Expression of the antiapoptosis gene, AAC-11, as a prognosis marker in non-small cell lung cancer. Lung Cancer (Amsterdam, The Netherlands) 2001; 34: 53–57. 10.1016/s0169-5002(01)00213-6 11557113

[bib22] Kim JW, Cho HS, Kim JH, Hur SY, Kim TE, Lee JM et al. AAC-11 overexpression induces invasion and protects cervical cancer cells from apoptosis. Lab Invest 2000; 80: 587–594. 10780674 10.1038/labinvest.3780063

[bib23] Song KH, Kim SH, Noh KH, Bae HC, Kim JH, Lee HJ et al. Apoptosis inhibitor 5 increases metastasis via Erk-mediated MMP expression. BMB Rep 2015; 48: 330–335. 25248562 10.5483/BMBRep.2015.48.6.139 PMC4578619

[bib24] Garcia-Jove Navarro M, Basset C, Arcondeguy T, Touriol C, Perez G, Prats H et al. Api5 contributes to E2F1 control of the G1/S cell cycle phase transition. PLoS ONE 2013; 8: e71443. 23940755 10.1371/journal.pone.0071443 PMC3737092

[bib25] Xu C, Fan ZP, Muller P, Fogley R, DiBiase A, Trompouki E et al. Nanog-like regulates endoderm formation through the Mxtx2-Nodal pathway. Dev Cell 2012; 22: 625–638. 22421047 10.1016/j.devcel.2012.01.003 PMC3319042

[bib26] Herbert C, Schieborr U, Saxena K, Juraszek J, De Smet F, Alcouffe C et al. Molecular mechanism of SSR128129E, an extracellularly acting, small-molecule, allosteric inhibitor of FGF receptor signaling. Cancer Cell 2013; 23: 489–501. 23597563 10.1016/j.ccr.2013.02.018

[bib27] Bono F, De Smet F, Herbert C, De Bock K, Georgiadou M, Fons P et al. Inhibition of tumor angiogenesis and growth by a small-molecule multi-FGF receptor blocker with allosteric properties. Cancer Cell 2013; 23: 477–488. 23597562 10.1016/j.ccr.2013.02.019

[bib28] Kwan KM, Fujimoto E, Grabher C, Mangum BD, Hardy ME, Campbell DS et al. The Tol2kit: a multisite gateway-based construction kit for Tol2 transposon transgenesis constructs. Dev Dyn 2007; 236: 3088–3099. 17937395 10.1002/dvdy.21343

[bib29] Han HD, Song CK, Park YS, Noh KH, Kim JH, Hwang T et al. A chitosan hydrogel-based cancer drug delivery system exhibits synergistic antitumor effects by combining with a vaccinia viral vaccine. Int J Pharmaceutics 2008; 350: 27–34. 10.1016/j.ijpharm.2007.08.014 17897800

[bib30] Westerfield M. The Zebrafish Book: A Guide for the Laboratory Use of Zebrafish (Danio Rerio) 5th edn. Institute of Neuroscience, University of Oregon: Eugene, OR, USA, 2007.

[bib31] Kimmel CB, Ballard WW, Kimmel SR, Ullmann B, Schilling TF. Stages of embryonic development of the zebrafish. Dev Dyn 1995; 203: 253–310. 8589427 10.1002/aja.1002030302

[bib32] Brend T, Holley SA. Zebrafish whole mount high-resolution double fluorescent *in situ* hybridization. J Visualized Exp 2009; 25: e1229. 10.3791/1229 PMC2789764 19322135

