# Supplementary material for: API5 confers cancer stem cell-like properties through the FGF2-NANOG axis
Source: Oncogenesis. 2017 Jan 16;6(1):e285–. doi: 10.1038/oncsis.2016.87 (PMC5294250; doi:10.1038/oncsis.2016.87)

**SUPPLEMENTARY INFORMATION**

**API5 confers cancer stem cell-like properties through the FGF2-NANOG axis**

Kwon-Ho Song, Hanbyoul Cho, Suhyun Kim, Hyo-Jung Lee, Se Jin Oh, Seon Rang Woo, Soon-Oh Hong, Han Sol Jang, Kyung Hee Noh, Chel Hun Choi, Joon-Yong Chung, Stephen M. Hewitt, Jae-Hoon Kim, Minjoo Son, Seok-Ho Kim, Byung Il Lee, Hae-Chul Park, Young-Ki Bae, and Tae Woo Kim

**Table S1**. Univariate and multivariate analyses of the associations between prognostic variables and overall survival in cervical cancer.

**Figure S1.** FGF2 silencing decreased the sphere-forming capacity induced by API5.

**Figure S2.** Ectopic expression of *api5* induces the expression of stem cell markers in vivo.

**Figure S3.** FGF2 expression in patients with cervical cancer.

**Table S1**. Univariate and multivariate analyses of the associations between prognostic variables and overall survival in cervical cancer.

|  | Overall Survival hazard ratio [95% CI], *p* value | | |
| --- | --- | --- | --- |
|  | Univariate |  | Multivariate |
| FIGO stage (II-IV) | 3.17 [1.22-8.25], 0.018* |  | 3.08 [1.12-8.45], 0.028* |
| Tumor grade (poor) | 2.56 [0.97-6.75], 0.056 |  | NA |
| Cell type (non-SCC) | 3.27 [1.24-8.60], 0.016* |  | 2.87 [1.07-7.64], 0.035* |
| Tumor size (> 4 cm) | 2.00 [0.76-5.27], 0.158 |  | NA |
| LN metastasis | 1.36 [0.35-6.08], 0.686 |  | NA |
| Age | 0.62 [0.22-1.77], 0.376 |  | NA |
| SCC+ | 1.75 [0.60-5.05], 0.300 |  | NA |
| FGF2+ | 2.24 [0.83-6.06], 0.109 |  | NA |
| API5+/FGF2+ | 4.10 [1.55-10.85], 0.004* |  | 2.93 [1.04-8.21], 0.041* |
| API5+/NANOG+ | 8.19 [3.11-21.55], <0.001* |  | 6.39 [2.32-17.58], <0.001* |
| API5+/NANOG+/FGF2+ | 8.69 [3.14-24.03], <0.001* |  | 5.78 [1.73-19.27], 0.004* |

Abbreviations: CI, confidence interval; FIGO, International Federation of Gynecology and Obstetrics; LN, lymph node; NA, not applicable; SCC, squamous cell carcinoma. **p* < 0.05.

**SUPPLEMENTARY FIGURE LEGENDS**

**Figure S1.** FGF2 silencing decreased the sphere-forming capacity induced by API5. CaSki-API5, CUMC6-API5 and HEK293-API5 cells were transfected with siGFP or siFGF2. *In vitro* tumor sphere forming assay in low density suspension cultures (1000cells/well). Error bars represent mean ± SD. Data presented are representative of three independent experiments.

**Figure S2.** Ectopic expression of *api5* induces the expression of stem cell markers in vivo. All images are lateral views of the spinal cord of zebrafish embryo, with anterior to the left and dorsal to the top. Zebrafish embryos were heat shocked at 22 hpf after injection of *egfp* or *api5-egfp* DNA. All embryos were collected at 24 hpf and labeled by fluorescent in situ RNA hybridization with *sox2* (**a**), *oct4* (**b**) and *klf4* (**c**) probes (red color). Graph shows quantification of the number of EGFP+Cy3+ cells (a: p< 0.01, b: p<0.01, c: p<0.05 each respectively). Data were obtained from each of the 6 larvae. Scale bars:50 μm.

**Figure S3.** FGF2 expression in patients with cervical cancer. (**a**) Box plot depiction of immunohistochemical staining data. The histoscores were computed based on intensity and tissue area of positive staining. In box-and-whisker plots, horizontal bars indicate the medians, boxes indicate 25th to 75th percentiles, and whiskers indicate the lowest and highest datum within the 1.5 interquartile range of the lower and upper quartiles, respectively. Symbols indicate individual samples. Numbers associated with symbols indicate case numbers. LGCIN, low-grade CIN; HGCIN, high-grade CIN. (**b**) Overall survival curves for ovarian cancer patients according to FGF2 expression.


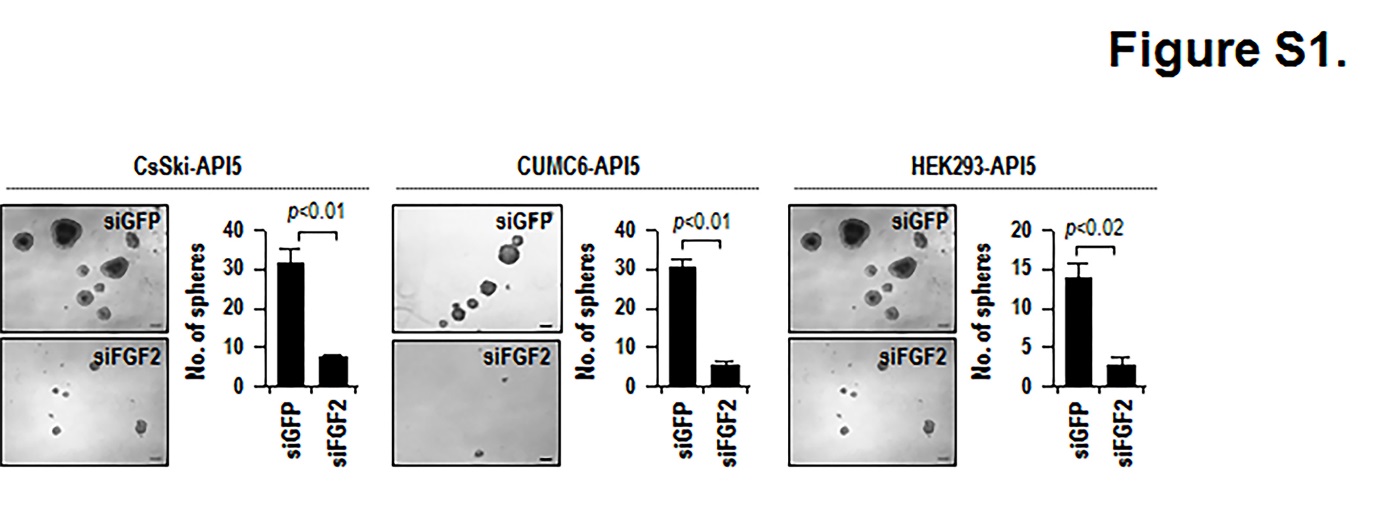


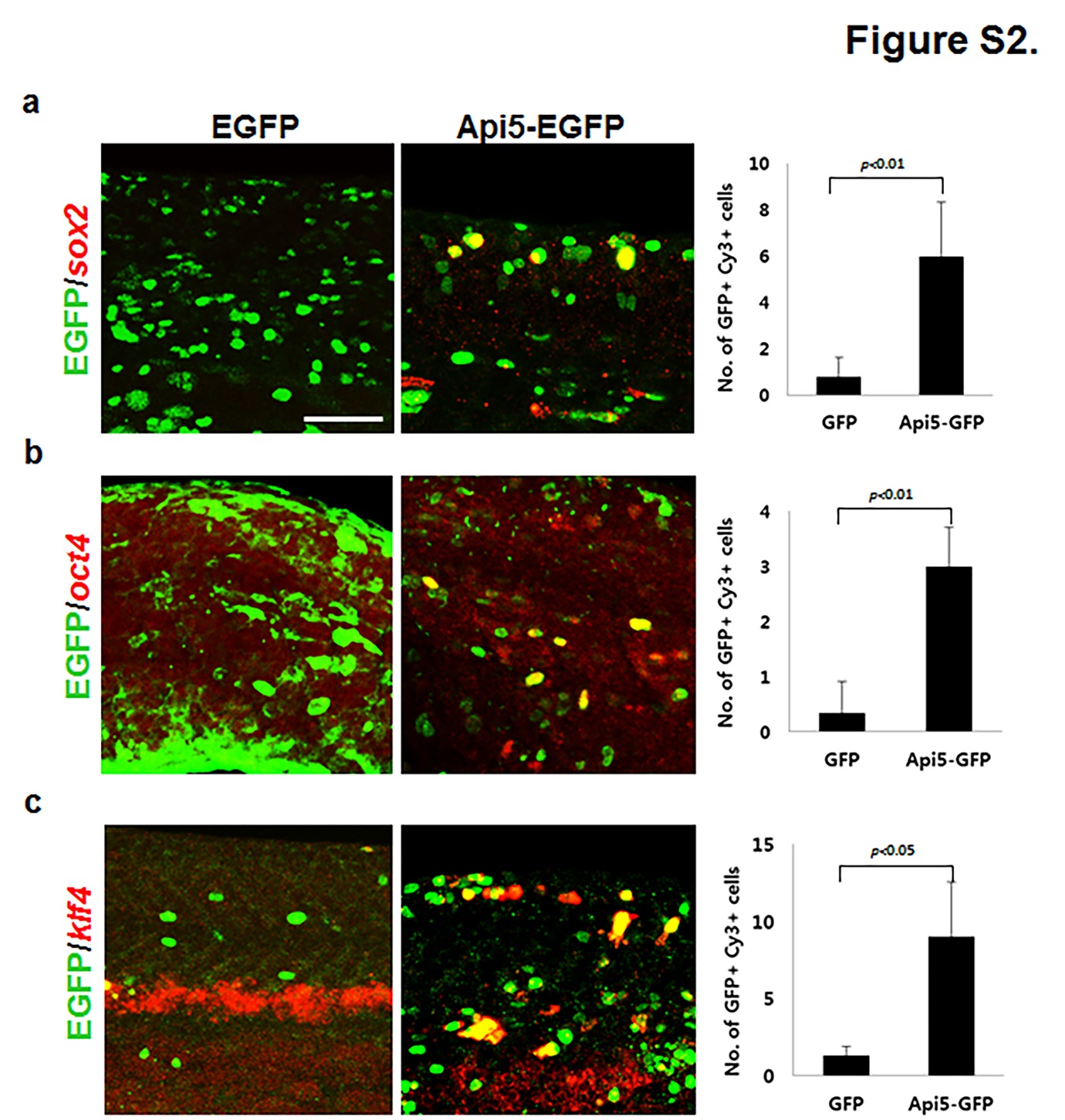


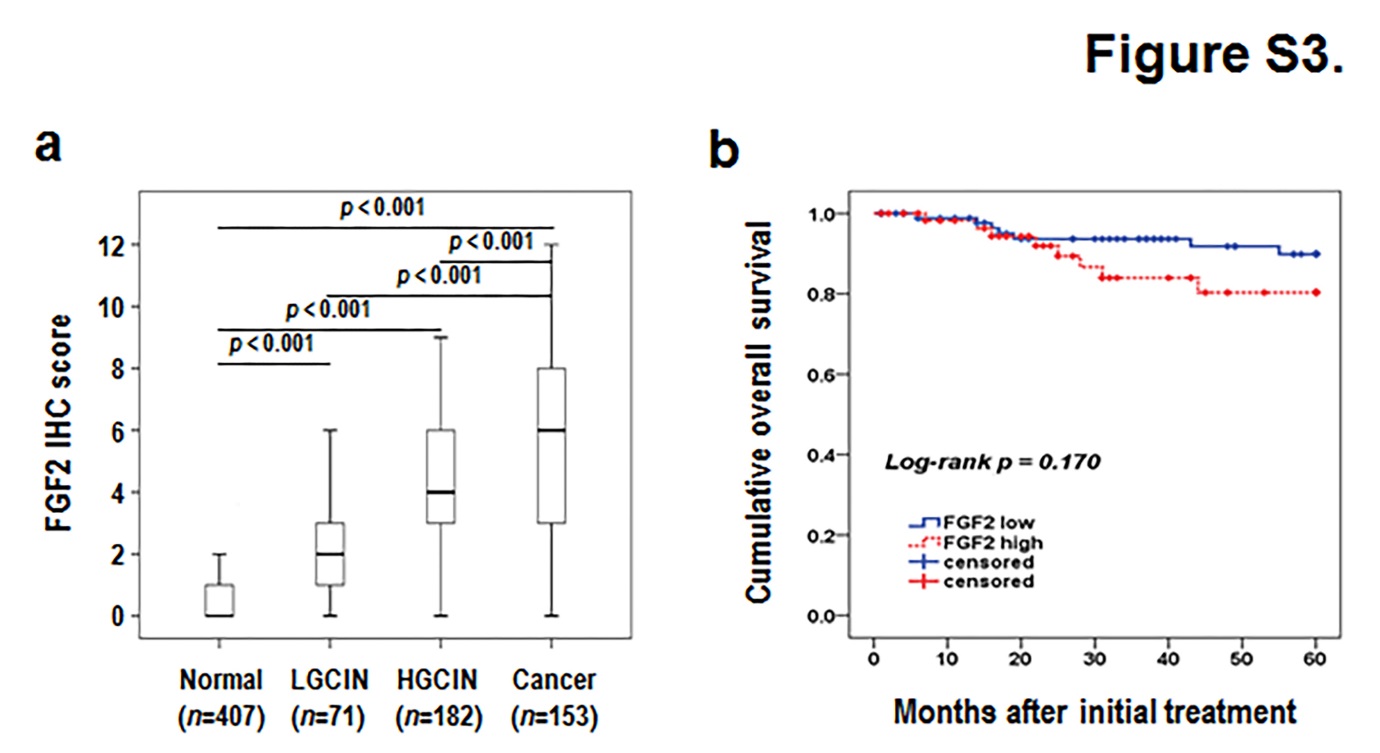

Supplement: Supplementary Information [file oncsis201687x1.docx]
